# Treatment of malignant peripheral nerve sheath tumors in pediatric NF1 disease

**DOI:** 10.1007/s00381-020-04687-3

**Published:** 2020-06-03

**Authors:** Enrico Martin, Uta E. Flucke, J. Henk Coert, Max M. van Noesel

**Affiliations:** 1 Department of Plastic and Reconstructive Surgery, University Medical Center Utrecht, G04.126, PO Box 85060, 3508 AB Utrecht, the Netherlands; 2 Department of Solid Tumors, Princess Máxima Center for Pediatric Oncology, Utrecht, the Netherlands; 3 Department of Pathology, Radboud University Medical Center, Nijmegen, the Netherlands

**Keywords:** Emerging therapies, MPNST, NF1, Radiotherapy, Surgical treatment, Systemic treatment

## Abstract

**Background:**

Malignant peripheral nerve sheath tumors (MPNSTs) are rare yet highly aggressive soft tissue sarcomas. Children with neurofibromatosis type 1 (NF1) have a 10% lifetime risk for development of MPNST. Prognosis remains poor and survival seems worse for NF1 patients.

**Methods:**

This narrative review highlights current practices and pitfalls in the management of MPNST in pediatric NF1 patients.

**Results:**

Preoperative diagnostics can be challenging, but PET scans have shown to be useful tools. More recently, functional MRI holds promise as well. Surgery remains the mainstay treatment for these patients, but careful planning is needed to minimize postoperative morbidity. Functional reconstructions can play a role in improving functional status. Radiotherapy can be administered to enhance local control in selected cases, but care should be taken to minimize radiation effects as well as reduce the risk of secondary malignancies. The exact role of chemotherapy has yet to be determined. Reports on the efficacy of chemotherapy vary as some report lower effects in NF1 populations. Promisingly, survival seems to ameliorate in the last few decades and response rates of chemotherapy may increase in NF1 populations when administering it as part of standard of care. However, in metastasized disease, response rates remain poor. New systemic therapies are therefore desperately warranted and multiple trials are currently investigating the role of drugs. Targeted drugs are nevertheless not yet included in first line treatment.

**Conclusion:**

Both research and clinical efforts benefit from multidisciplinary approaches with international collaborations in this rare malignancy.

## Introduction

Children with neurofibromatosis type I (NF1) are at an increased risk of developing multiple tumors including neurofibromas as a result of neurofibromin inhibition involved in the Ras pathway [1]. NF1 disease is a frequent genetic disease, affecting 1 in 3000 to 5000 people. The cumulative life time risk of developing malignant peripheral nerve sheath tumors (MPNST) is 8–13% [2–5]. MPNSTs are rare in the general population encompassing only 2–4% of all soft tissue sarcomas [6]. Less than 10% of those patients are children [7, 8], but MPNST is the third most common non-rhabdomyosarcoma STS (NRSTS) in children [9]. Of all MPNSTs, almost 40–50% of patients are NF1 patients; the remaining 50–60% develop sporadically or are radiation-induced [10–15].

Genetically, NF1 is characterized by the loss of heterozygosity of the *NF1* tumor suppressor gene, although not in all patients [16]. Loss of *NF1* function leads to activation of the Ras pathway and upregulation of mitogen-activated protein kinase (MAPK) and phosphoinositide 3-kinase (PI3K) leading to cell proliferation [17]. NF1 patients develop neurofibromas, plexiform neurofibromas, in a nerve plexus lateral of the vertebrae and sacrum. For malignant transformation, loss of neurofibromin alone is not enough to develop an MPNST [18]. Although the landscape of tumor drivers in MPNST is not clear yet, MPNST are characterized by extensive genetic instability with several frequently observed genetic alterations considered as tumor driver events. The most frequent events are amplification of receptor tyrosine kinases and mutations of the genes *CDKN2A/p16*, *SUZ12*, *TP53*, and *PTEN*. One or more are considered necessary in the development of an MPNST [19–24].

The cornerstone of treatment in MPNST remains surgical excision, which is strongly associated with improved survival. However, MPNSTs have been reported initially unresectable in 17–53% of children, which is higher than any other pediatric NRSTS [25–29]. Radiotherapy and chemotherapy are incorporated in the treatment of unresectable or large, localized pediatric MPNST [29]. Despite curative intents of treatment, survival remains relatively poor with 5-year survival rates of 51–66% [8, 25, 29]. Promisingly, survival has improved over the past decade in pediatric MPNST [8]. And children have a superior survival in comparison with adult patients [7, 8]. Although the influence of NF1 on survival is strongly debated in adults [30], in pediatric series, it has repeatedly been shown an independent predictor of survival [8, 25, 29, 31]. Importantly, NF1 children tend to present with larger tumors than non-NF1 patients, and the outcome is still poor and unsatisfactory. There is a need for novel treatment strategies and compounds.

This review will discuss the surgical approach to these tumors, current treatment recommendations, and emerging therapeutic options in pediatric NF1–associated MPNST.

## Genomic landscape

NF1 disease is based on a germline mutation in the *NF1* gene on chromosome 17q11.2. The *NF1* gene is large, spanning 350 kb of genomic DNA and encoding 11–13 kb mRNA containing 59 exons, and results in a 320-kDa neurofibromin protein. Neurofibromin downregulates the Ras protein, and frequent germ line or somatic mutations are present in several human cancers. Therefore, *NF1* is considered a tumor suppressor gene. More than 500 mutations in the *NF1* gene have been identified. Mostly, they result in truncation of the neurofibromin protein and loss of function [32]. The development of neurofibromas result as a loss of a second allele, although this cannot be established in all neurofibromas, possibly due to the complex and large *NF1* gene or to the existence of other, so far unknown genes involved in a second hit [16]. The understanding of the genomic landscape of MPNST in NF-related and non-NF1-related disease is complex and incomplete. A number of recurrent pathological events are recognized, lacking a consistent pattern among individuals and tumors. It includes recurrent loss of “classical” tumor suppressor genes *NF1*, *TP53*, *CDKN2A*/*p16* and more recently also mutations in PRC2 complex genes [20, 33]. The PRC2 complex consists of chromatin-modifying proteins involved in epigenetic suppression of gene expression. In MPNST, loss of function mutations have been observed in *SUZ12* and *EED* in a mutually exclusive manner, and also in regulators of the PRC2 complex, such as EPC1, CHD4, AEBP2, and ATRX [24]. In addition, it should be noted that there is considerable intra-tumor heterogeneity, complicating our understanding of tumor driver events in MPNST [34].

## Diagnostics of MPNST

Whenever a malignant tumor arises from a peripheral nerve or within a pre-existent neurofibroma, diagnostic criteria are fulfilled for an MPNST. However there is still lack of standardized pathology diagnostic criteria outside these settings. Cells involved may resemble Schwann cells, perineurial cells, and fibroblasts; tumors can therefore morphologically be heterogeneous. Classically, MPNSTs consist of monomorphic spindle cells arranged in long fascicles. Due to alternating cellularity, a marble-like aspect is commonly given. Perivascular cuffs are a typical feature. Nuclei are often wavy or buckled, and mitotic figures may be numerous. The cytoplasm is inconspicuous. When arising in a neurofibroma, high cellularity with fascicular arrangement and mitotic figures make the diagnosis of an MPNST. However, there is a broad morphological spectrum ranging from spindle cell (most common) to epithelioid and pleomorphic histology [35, 36]. Immunohistochemistry shows variable features with incomplete staining of S100 and SOX10 in up to 85% of the cases, in contrast to melanoma and schwannoma that are commonly strongly positive for these markers. However, very importantly, when these markers are absent, an MPNST cannot be ruled out. CD34 and EMA may be positive but they are unspecific and therefore not very helpful. MDM2 is expressed in a subset of tumors often NF1 related, but there is no amplification of the corresponding gene as seen in dedifferentiated liposarcoma [35–37]. Loss of H3K27me3 due to mutational inactivation of the PRC2 complex has been observed in a subset of MPNST showing often high-grade morphology and an association with previous radiation therapy or sporadic occurrence. In NF1-related tumors, this protein is aberrant to a lesser extent. Neurofibromas with or without atypia are reported to have a retained protein, which can aid in the distinction from MPNST [38]. Methylation profiling seems to be a reliable molecular technique to differentiate biologically relevant groups of peripheral nerve sheath tumors as well [39].

### Imaging of MPNST

In NF1 patients, the distinction between (plexiform) neurofibroma and MPNST is complex. They may both present with similar symptoms, making it commonly impossible to differentiate between benign and malignant lesions based just on clinical presentation [40, 41]. Imaging plays an important role in diagnostic work-up, as biopsies are cumbersome in NF1 patients especially since they may be painful and result in persisting nerve damage [42]. Additionally, in NF1 patients, sampling errors are common [43, 44]. Magnetic resonance imaging (MRI) is widely used but may be inadequate to detect and especially distinguish benign and malignant lesions [45, 46]. Although the presence of a target sign, a hypointense region on T2-weighted images, represents a benign lesion with great likelihood, its absence is unspecific [47]. The use of ^18^F-fluorodeoxyglucose positron emission tomography/computed tomography (^18^F-FDG PET-CT) has gained popularity in NF1 patients to detect MPNSTs. Several studies suggest a high sensitivity of up to 95% to detect MPNSTs using the maximum standardized uptake values (SUVmax) [45, 48, 49]. However, while the most commonly used threshold is ≥ 3.5, an ideal threshold is lacking as some studies suggest high false positive rates using this threshold [50, 51]. Numerous studies have tried to find parameters with higher accuracy and reproducibility to detect MPNSTs. This includes the use of the SUVmax to liver SUVmean or SUVmax (T/L ratio) which generally results in equal accuracy as SUVmax [52–54]. Thresholds vary between 1.5 and 3.0, and an ideal threshold has yet to be established. The use of T/L ratio does however diminish measurement variations between scanners. Delayed scanning at 4 h has been proposed as well and may increase specificity, but requires more resources and exposes patients to additional radiation [55]. More recently, functional MRI sequences including diffusion-weighted imaging (DWI) and apparent diffusion coefficient (ADC) have shown promising results [50, 56]. Because MPNSTs show increased cellularity, minimal ADC values of < 1 × 10^−3^ may be useful. Reported sensitivity ranged between 89 and 98% and specificity 93 and 94%. Further research is however necessary to establish their role in the diagnostic work-up of MPNST. The use of PET-MRI scans in NF1 populations is attractive as it combines the accuracy of both modalities and diminishes radiation exposure [46, 51].

### Liquid biopsies

Liquid biopsies have established an important role in detection and treatment of numerous cancers over the past decades. These include breast cancer [57], prostate cancer [58], ,and lung cancer [59], but they have not yet been widely used in NF1 and sarcomas. To date, three studies have been published on liquid biopsies, reporting the utility of four circulating biomarkers. One study found elevated levels of adrenomedullin (ADM) as a potential biomarker of malignant transformation among NF1 patients with and without MPNST [60]. Concentrations of ADM were higher among patients with MPNST compared with those with only plexiform neurofibromas (0.24 vs 0.18 ng/mL, *p* = 0.03). Another study reported soluble AXL (sAXL) to be higher among NF1 patients with plexiform neurofibromas and MPNSTs compared with NF1 patients with dermal neurofibromas only [61]. But sAXL could not differentiate MPNST patients from those with plexiform neurofibromas only. The third study found insulin growth factor-binding protein 1 (IGFBP1) elevated in MPNST patients compared with NF1 control patients [62]. IGFBP1 levels were able to detect MPNST with 90% sensitivity and 50% specificity. The same study also found elevated levels of regulated upon activated normal T-cell expressed and secreted (RANTES) to detect MPNST with 90% sensitivity and 26% specificity.

### Staging of MPNST

MPNSTs are present in 5–11% of cases with synchronous metastases [13, 15, 63]. Metastatic sites of MPNST are similar to other STS and usually involve the lungs. They may also metastasize to the bone and liver, and in rare occasions to the regional lymph nodes and even the brain [63, 64]. Staging of MPNSTs therefore requires at least preoperative lung CT scanning which may be accompanied with an ^18^FDG-PET scan. Other staging modalities may be used in selected cases but generally do not play a role in MPNST work-up. Avidity of other lesions than the MPNST on ^18^FDG-PET scan may however require further investigation. A sentinel node procedure should not routinely be employed, because of the low probability of lymph node involvement.

## Surgical management

### Surgical margins

Ideally, MPNSTs should be removed with clear margins in order to minimize local recurrence rates and optimize survival, making it the routine treatment of choice [11, 13, 65, 66]. Postoperative morbidity is however high when resecting MPNSTs, especially when they originate from major nerves or in the brachial or sacral plexus. Contrarily, conventional and even atypical neurofibromas can be excised using nerve-sparing surgery resulting in minimal morbidity yet with low recurrence rates [67]. It is therefore important to have a reliable diagnosis before surgically excising a nerve sheath tumor in NF1 patients, as unnecessary function loss may be avoided in a patient cohort that is already prone to disabilities. Nonetheless, even when obtaining microscopic-free margins (R0), MPNSTs can recur [10–12, 68, 69]. Some argue that this may be due to its perineural origin, and microscopic skip lesions may be present along the nerve [70]. In a recent survey, several surgeons would advocate possibly resecting more of the nerve of origin, but this has not been studied in a clinical setting [71]. Fresh frozen coupes may be indicated intraoperatively to ascertain complete resection in nerve endings [10, 12, 70]. However, several large MPNST cohorts in adults have shown that microscopic-positive margins do not impair survival [11, 13, 15]. It has also been shown that close margin surgery, including epineural dissection of adjacent nerves, in combination with radiotherapy does not significantly increase recurrence rates in STS [72, 73]. This indicates that MPNSTs arising from a plexus could initially be treated with en bloc resection of its originating nerve, yet nerves that are not completely encompassed by tumor may be spared. Such a surgical approach may obviate the need for a forequarter amputation in some children, which is important to bear in mind when children may survive well beyond 5–10 years [8, 29].

### Reconstructions

Resection of functional nerves is sometimes inevitable and postoperative functional deficits are reported in 30–40% of MPNST patients [74]. Especially when survival is slowly increasing for pediatric patients, preventive or counter-acting measures should be taken into consideration. To date, functional reconstructions, reconstructions aimed at restoring lost function, are only rarely performed in any STS [75, 76]. Reasons for this include the hesitance of surgeons to perform such operations, not knowing the possibilities for reconstruction, and the lack of literature on indications and outcome as the focus of research has been on improving oncological outcomes [76]. Nonetheless, research has shown that good functional outcomes can generally be expected despite the use of radiotherapy and chemotherapy, even in nerve reconstructions [76–78]. A recent unpublished survey among several surgical subspecialties involved in MPNST treatment showed that there is interest in performing functional reconstructions in MPNST patients, but surgical oncologists were least likely to consider such reconstructions even though they operated most patients. Regardless of subspecialty, respondents reported a minimal prognosis of 3 years or more before functional reconstructions should be considered. With an increasing knowledge of the use of (acute) nerve transfers in trauma and brachial plexus birth palsy, these techniques could be incorporated in the treatment of MPNSTs as well. Especially, since children are known to experience better results after nerve reconstruction than adults [79]. Moreover, distal nerve transfers may facilitate reconstruction outside of an irradiated field and diminish time to restoration of function. Other options to restore function also include tendon transfers or grafting as well as innervated (free) muscle transfers or cutaneous flaps. Exact choice for reconstruction relies on multiple factors such as tumor location, defect size, and need for coverage of soft tissue defects. Combining the knowledge of reconstructive possibilities by nerve and reconstructive surgeons as an extension to oncological resections may therefore improve the delicate balance between oncological and functional outcomes. Discussions regarding function preservation must be held before initial surgery and be weighed in when considering prognosis and the use of radiotherapy. The use of free flaps may for instance diminish postoperative complications when radiotherapy is administered [80, 81]. Future research should be encouraged to explore the field of functional status after MPNSTs in children, and define indications and guidelines to incorporate functional reconstructions as part of the surgical treatment plan.

## Radiotherapy

The exact use of radiotherapy in MPNST is controversial, even more so in pediatric patients, as improvement of oncological outcomes needs to be considered in light of potential side effects. Radiotherapy is administered to increase local control of MPNST, but has not shown to affect overall survival (OS) [11, 15, 82]. The dosage of radiotherapy usually followed adult STS guidelines that recommended 60–66 Gy in a postoperative setting or 50 Gy preoperatively for tumors > 5 cm and positive margins [82–84]. In adults, it has shown that preoperative administration reduces long-term fibrosis and therefore enhances limb function, albeit a higher rate of postoperative complications [85, 86]. In children, several studies try to limit the dose of irradiation for STS. Radiation treatment is known to stunt growth of involved tissues and a reduced dose may be beneficial in decreasing side effects. However, this reduced dose is not based on randomized trials and it may limit the effectiveness of irradiation. In the European paediatric Soft Tissue Sarcoma Study Group (EpSSG), irradiation doses for NRSTS ranged from 50.4–54 Gy for tumors of high grade (G2 or 3), size > 5 cm, or irresectable tumors [29]. In a recent nationwide series of pediatric MPNST in the Netherlands including 70 patients, radiotherapy was administered in 37.5%, with no difference between NF1 and sporadic patients [8]. Preoperative radiotherapy was applied in 20% of children receiving radiotherapy. Although both pediatric studies could not confirm the additive role of radiotherapy in pediatric MPNST, based on the adult results with a higher treatment dose, it is advised to apply radiation in pediatric MPNST also [13].

## Chemotherapy

The role of chemotherapy in MPNST is limited, especially in NF1-associated MPNST and has not been fully established [9, 27, 87–90]. In advanced MPNST disease where it is estimated that negative margins cannot be achieved by surgery alone, (neo) adjuvant chemotherapy and radiotherapy are applied. In adult studies, the most established chemotherapeutic drugs in MPNST and STS patients are ifosfamide and doxorubicin, alone or in combination [91]. In pediatric NRSTS, various chemotherapy combinations are used, including vincristine, cyclophosphamide, ifosfamide, doxorubicin, and dactinomycin [27, 89]. The most recent pediatric MPNST study used the combination ifosfamide and doxorubicin, in analogy to the most used combination in adults [29]. In a recent adult analysis, the chemotherapy response varied between 17.9 (NF1 related) and 44.4% (non-NF1 related) [92]. Two pediatric studies studied chemotherapy response in (non) NF1 disease. In a German-Italian study including 167 pediatric MPNST patients, the response to chemotherapy was significantly lower in NF1 in comparison with non-NF1 patients, i.e., 17.6% and 55.3%, respectively [25]. In the EpSSG study including 26 evaluable chemotherapy patients, the chemotherapy response was 46.2% with no difference between NF1 (40%) and non-NF1 patients (50%) [29]. The role of chemotherapy in pediatric MPNST is mostly for children with unresectable disease providing moderate chemotherapy response, and most data indicate a lesser chemotherapy responsiveness in children with NF1 disease.

## Survival

In the large meta-analysis of American and European pediatric NRSTS, NF1-associated MPNST was the most unfavorable STS in children. The OS for all STS was 60.0% and 51.5% at 5 and 10 years, respectively. For MPNST-NF1 the 5-year OS was 11.1% (3.8–32.3%), while the 5-year OS in the MPNST non-NF1 group was 44.7% (32.1–62.3%) [9]. Earlier studies reported an outcome of 5-year OS of 55.1% for non-NF1 pediatric MPNST and 32.1% for NF1 MPNST in a cohort of 167 pediatric patients [25]. Two more recent cohorts of patients showed ameliorated survival outcomes. In a cohort of 51 patients, the 5-year OS for localized non-NF1 MPNST was 80% and 42.6% for NF1-related MPNST [29]. Another study reported 5-year OS of 52.4% for NF1-related MPNST and 75.8% for non-NF1-related MPNST [8]. These results are relatively favorable compared with outcome in adult patients with MPNST. In most mentioned pediatric studies, chemotherapy was included in the standard of care for large or advanced MPNST and seems to indicate a role in the systemic therapy in the treatment of pediatric MPNST. For metastatic and recurrent disease in both adult and pediatric patients, the results of treatment are extremely poor and there is a need for other treatment strategies in MPNST.

## Emerging therapeutic options

Considering the biology of MPNST tumors, obvious and potential targets can be recognized. Growth and activation receptors, such as vascular endothelial growth factor receptors (VEGFR), epidermal GFR (EGFR), and platelet-derived GFR (PDGFR), activate two pivotal intracellular activation pathways, i.e., the MAPK signal transduction pathway and the P13K-AKT-mammalian target of rapamycin (mTOR). Potential targets for therapy are found on all three levels of normal signal transduction, i.e., receptor kinases, MAPK pathway, and the P13K-AKT-mTOR pathway. Currently, the most considered in NF1 disease is inhibition of the MAPK pathway via MEK inhibitors (MEKi). MEKi potentially neutralize the loss of *NF1* in controlling Ras activation. Selumetinib is a MEK1/2 inhibitor therefore can convey an anti-tumor signal by inhibiting Ras signals. In pediatric NF1 patients, selumetinib was very effective in controlling the growth of inoperable plexiform neurofibromas. Partial response was observed in 17/24 patients (71%) [93]. Selumetinib was consequently approved in 2019 by the US FDA for treatment of pediatric patients (> 3 years) with symptomatic or inoperable plexiform neurofibroma. In the treatment of MPNST, selumetinib has not yet been approved, but several trials including (plexiform) neurofibromas and some including MPNST are being conducted (i.e., NCT03433183, NCT02124772). Targeting of upstream tyrosine kinase receptors, alone or in combination with chemotherapy, can also de-activate the MAPK or mTOR pathways. A phase 2 randomized study of doxorubicin plus olaratumab (anti-PDGFRα antibody) compared with doxorubicin monotherapy showed a better OS and trend towards improved median progression-free survival for the combination treatment arm [94]. The study included MPNST patients but did not report separate analyses of this subgroup. Imatinib inhibits specifically the tyrosine kinase domain in ABL (effective in BCR-ABL translocated leukemia), c-KIT, and PDGFR. Imatinib has proven activity in other (pediatric) STS, including dermatofibrosarcoma protuberans (DFSP), gastrointestinal stromal tumor (GIST), and desmoid fibromatosis. However, in MPNST, imatinib has not been found effective [95, 96]. Other RTK inhibitors, such as erlotinib, sunitinib, sorafenib, cediranib, and dasatinib, are all potential inhibitors of NF1-related tumors but have not been found effective in few phase 1 studies with limited information on effectiveness, and they all reported on considerable side effects [97–102]. Inhibition of the mTOR pathway has been effective in several tumor models [103]. One trial in 25 adult MPNST patients administered everolimus and bevacizumab combination therapy, but found only stable disease in 3 patients [104]. Currently, several combination therapies with mTOR inhibition are being undertaken including at least in some part of MPNST patients (NCT02584647, NCT01661283, NCT03433183, NCT02008877, NCT02601209). Anti-GD2-based immunotherapy could potentially be useful in MPNST. Gangliosides are abundantly expressed in neuro-ectodermal-derived tissues (Schwann cells) and tumors. In neurofibromas, the expression of gangliosides has been established [105]. For MPNST, this has not yet been studied. The CH(O)14,18 chimeric mouse-human anti-GD2 antibody has proven to be safe and effective in pediatric high risk neuroblastoma tumors resulting in significantly increased OS [106]. Dinutuximab has been FDA-approved and dinutuximab-beta has been EMA-approved for standard use in high risk neuroblastoma. So far, no studies applying anti-GD2 antibodies in MPNST or other neuro-ectodermal tumors (melanoma, non-small cell lung cancer) have been conducted. Another type of immunotherapy using anti-CTLA4 and/or anti-PD(L)-1 inhibitors has proven effectiveness in several tumors (melanoma, non-small cell lung cancer, mesothelioma and others). A recent phase 2 trial combining nivolumab with ipilimumab in advanced sarcomas yielded promising effects, although the study did not specify the included types of sarcomas [107]. One trial is currently ongoing in MPNSTs specifically investigating the effect of pembrolizumab (NCT02691026). Oncolytic viruses have proven effective in vivo, and one trial is currently investigating the effect of oncolytic measles virus in MPNST patients (NCT02700230) [103].

## Conclusion

Pediatric NF1 patients have a 10% lifetime risk–developing MPNST. MPNSTs are genetically unstable tumors with multiple genetic alterations superimposed on the germ line NF1 genetic defect. MPNSTs are relatively chemo-insensitive tumors for which the mainstay of treatment is still resection with tumor-free margins. We eluded novel possibilities to use reconstructive techniques such as nerve reconstructions to increase postoperative function without compromising survival. Adjuvant radiotherapy is important in establishing local control in R1 resections. However, the *NF1* deficiency carries a risk for increased secondary tumors later in life. Many novel and potential compounds aimed at the molecular pathways involved in growth and development of MPNST and neurofibromas have been recognized. Considering the rarity of MPNST, for future trials, it is important to combine MPNST patients with patients with plexiform neurofibroma or in combination with other sarcomas (basket trials). Advancing the future of NF- affected individuals is important and challenging.

## Data Availability

Not applicable

## References

[1] Basu TN, Gutmann DH, Fletcher JA, Glover TW, Collins FS, Downward J (1992). Aberrant regulation of ras proteins in malignant tumour cells from type 1 neurofibromatosis patients. Nature.

[2] Ducatman BS, Scheithauer BW, Piepgras DG, Reiman HM, Ilstrup DM (1986). Malignant peripheral nerve sheath tumors. A clinicopathologic study of 120 cases. Cancer.

[3] Evans DGR, Baser ME, McGaughran J, Sharif S, Howard E, Moran A (2002). Malignant peripheral nerve sheath tumours in neurofibromatosis 1. J Med Genet.

[4] Pasmant E, Sabbagh A, Spurlock G, Laurendeau I, Grillo E, Hamel MJ, Martin L, Barbarot S, Leheup B, Rodriguez D, Lacombe D, Dollfus H, Pasquier L, Isidor B, Ferkal S, Soulier J, Sanson M, Dieux-Coeslier A, Bièche I, Parfait B, Vidaud M, Wolkenstein P, Upadhyaya M, Vidaud D, members of the NF France Network (2010). NF1 microdeletions in neurofibromatosis type 1: from genotype to phenotype. Hum Mutat.

[5] Evans DGR, Huson SM, Birch JM (2012). Malignant peripheral nerve sheath tumours in inherited disease. Clin Sarcoma Res.

[6] Ng VY, Scharschmidt TJ, Mayerson JL, Fisher JL (2013). Incidence and survival in sarcoma in the United States: a focus on musculoskeletal lesions. Anticancer Res.

[7] Martin E, Muskens IS, Coert JH et al (2018) Treatment and survival differences across tumor sites in malignant peripheral nerve sheath tumors: a SEER database analysis. Neuro-Oncology Pract:1–10 10.1093/nop/npy025 PMC6656331 31386019

[8] Martin E, Coert JH, Flucke UE, Slooff WBM, de Sande MAJ, Noesel MM, Grünhagen DJ, Wijnen MHWA, Verhoef C (2019). Neurofibromatosis-associated malignant peripheral nerve sheath tumors in children have a worse prognosis: a nationwide cohort study. Pediatr Blood Cancer.

[9] Ferrari A, Miceli R, Rey A, Oberlin O, Orbach D, Brennan B, Mariani L, Carli M, Bisogno G, Cecchetto G, Salvo GLD, Casanova M, Vannoesel MM, Kelsey A, Stevens MC, Devidas M, Pappo AS, Spunt SL (2011). Non-metastatic unresected paediatric non-rhabdomyosarcoma soft tissue sarcomas: results of a pooled analysis from United States and European groups. Eur J Cancer.

[10] Zou C, Smith KD, Liu J, Lahat G, Myers S, Wang WL, Zhang W, McCutcheon IE, Slopis JM, Lazar AJ, Pollock RE, Lev D (2009). Clinical, pathological, and molecular variables predictive of malignant peripheral nerve sheath tumor outcome. Ann Surg.

[11] Stucky C-CH, Johnson KN, Gray RJ, Pockaj BA, Ocal IT, Rose PS, Wasif N (2012). Malignant peripheral nerve sheath tumors (MPNST): the Mayo Clinic experience. Ann Surg Oncol.

[12] Anghileri M, Miceli R, Fiore M, Mariani L, Ferrari A, Mussi C, Lozza L, Collini P, Olmi P, Casali PG, Pilotti S, Gronchi A (2006). Malignant peripheral nerve sheath tumors: prognostic factors and survival in a series of patients treated at a single institution. Cancer.

[13] Valentin T, Le Cesne A, Ray-Coquard I (2016). Management and prognosis of malignant peripheral nerve sheath tumors: the experience of the French Sarcoma Group (GSF-GETO). Eur J Cancer.

[14] Miao R, Wang H, Jacobson A, Lietz AP, Choy E, Raskin KA, Schwab JH, Deshpande V, Nielsen GP, DeLaney TF, Cote GM, Hornicek FJ, Chen YLE (2019). Radiation-induced and neurofibromatosis-associated malignant peripheral nerve sheath tumors (MPNST) have worse outcomes than sporadic MPNST. Radiother Oncol.

[15] Martin E, Coert JH, Flucke UE, Slooff WBM, Ho VKY, van der Graaf WT, van Dalen T, van de Sande MAJ, van Houdt WJ, Grünhagen DJ, Verhoef C (2019). A nationwide cohort study on treatment and survival in patients with malignant peripheral nerve sheath tumours. Eur J Cancer.

[16] Eisenbarth I, Beyer K, Krone W, Assum G (2000). Toward a survey of somatic mutation of the NF1 gene in benign neurofibromas of patients with neurofibromatosis type 1. Am J Hum Genet.

[17] Endo M, Yamamoto H, Setsu N, Kohashi K, Takahashi Y, Ishii T, Iida KI, Matsumoto Y, Hakozaki M, Aoki M, Iwasaki H, Dobashi Y, Nishiyama K, Iwamoto Y, Oda Y (2013). Prognostic significance of AKT/mTOR and MAPK pathways and antitumor effect of mTOR inhibitor in NF1-related and sporadic malignant peripheral nerve sheath tumors. Clin Cancer Res.

[18] Kluwe L, Friedrich RE, Mautner VF (1999). Allelic loss of the NF1 gene in NF1-associated plexiform neurofibromas. Cancer Genet Cytogenet.

[19] Beert E, Brems H, Daniels B (2011). Atypical neurofibromas in neurofibromatosis type 1 are premalignant tumors. Genes Chromosom Cancer.

[20] De Raedt T, Beert E, Pasmant E (2014). PRC2 loss amplifies Ras-driven transcription and confers sensitivity to BRD4-based therapies. Nature.

[21] Legius E, Dierick H, Wu R, Hall BK, Marynen P, Cassiman JJ, Glover TW (1994). TP53 mutations are frequent in malignant NF1 tumors. Genes Chromosom Cancer.

[22] Masliah-Planchon J, Pasmant E, Luscan A, Laurendeau I, Ortonne N, Hivelin M, Varin J, Valeyrie-Allanore L, Dumaine V, Lantieri L, Leroy K, Parfait B, Wolkenstein P, Vidaud M, Vidaud D, Bièche I (2013). MicroRNAome profiling in benign and malignant neurofibromatosis type 1-associated nerve sheath tumors: evidences of PTEN pathway alterations in early NF1 tumorigenesis. BMC Genomics.

[23] Upadhyaya M, Spurlock G, Thomas L, Thomas NST, Richards M, Mautner VF, Cooper DN, Guha A, Yan J (2012). Microarray-based copy number analysis of neurofibromatosis type-1 (NF1)-associated malignant peripheral nerve sheath tumors reveals a role for Rho-GTPase pathway genes in NF1 tumorigenesis. Hum Mutat.

[24] Zhang M, Wang Y, Jones S, Sausen M, McMahon K, Sharma R, Wang Q, Belzberg AJ, Chaichana K, Gallia GL, Gokaslan ZL, Riggins GJ, Wolinksy JP, Wood LD, Montgomery EA, Hruban RH, Kinzler KW, Papadopoulos N, Vogelstein B, Bettegowda C (2014). Somatic mutations of SUZ12 in malignant peripheral nerve sheath tumors. Nat Genet.

[25] Carli M, Ferrari A, Mattke A, Zanetti I, Casanova M, Bisogno G, Cecchetto G, Alaggio R, de Sio L, Koscielniak E, Sotti G, Treuner J (2005). Pediatric malignant peripheral nerve sheath tumor: the Italian and German soft tissue sarcoma cooperative group. J Clin Oncol.

[26] Pratt CB, Maurer HM, Gieser P, Salzberg A, Rao BN, Parham D, Thomas PRM, Marcus RB, Cantor A, Pick T, Green D, Neff J, Jenkins JJ (1998). Treatment of unresectable or metastatic pediatric soft tissue sarcomas with surgery, irradiation, and chemotherapy: a Pediatric Oncology Group study. Med Pediatr Oncol.

[27] Spunt SL, Hill DA, Motosue AM, Billups CA, Cain AM, Rao BN, Pratt CB, Merchant TE, Pappo AS (2002). Clinical features and outcome of initially unresected nonmetastatic pediatric nonrhabdomyosarcoma soft tissue sarcoma. J Clin Oncol.

[28] Ferrari A, Casanova M, Collini P, Meazza C, Luksch R, Massimino M, Cefalo G, Terenziani M, Spreafico F, Catania S, Gandola L, Gronchi A, Mariani L, Fossati-Bellani F (2005). Adult-type soft tissue sarcomas in pediatric-age patients: experience at the Istituto Nazionale Tumori in Milan. J Clin Oncol.

[29] van Noesel MM, Orbach D, Brennan B, Kelsey A, Zanetti I, de Salvo GL, Gaze MN, Craigie RJ, McHugh K, Francotte N, Collini P, Bisogno G, Casanova M, Ferrari A (2019). Outcome and prognostic factors in pediatric malignant peripheral nerve sheath tumors: an analysis of the European Pediatric Soft Tissue Sarcoma Group (EpSSG) NRSTS-2005 prospective study. Pediatr Blood Cancer.

[30] Kolberg M, Holand M, Agesen TH, Brekke HR, Liestol K, Hall KS, Mertens F, Picci P, Smeland S, Lothe RA (2013). Survival meta-analyses for >1800 malignant peripheral nerve sheath tumor patients with and without neurofibromatosis type 1. Neuro-Oncology.

[31] Meis JM, Enzinger FM, Martz KL, Neal JA (1992). Malignant peripheral nerve sheath tumors (malignant schwannomas) in children. Am J Surg Pathol.

[32] Upadhyaya M, Osborn MJ, Maynard J, Kim MR, Tamanoi F, Cooper DN (1997). Mutational and functional analysis of the neurofibromatosis type 1 (NF1) gene. Hum Genet.

[33] Lee W, Teckie S, Wiesner T, Ran L, Prieto Granada CN, Lin M, Zhu S, Cao Z, Liang Y, Sboner A, Tap WD, Fletcher JA, Huberman KH, Qin LX, Viale A, Singer S, Zheng D, Berger MF, Chen Y, Antonescu CR, Chi P (2014). PRC2 is recurrently inactivated through EED or SUZ12 loss in malignant peripheral nerve sheath tumors. Nat Genet.

[34] S.L. C (2016) The challenge of cancer genomics in rare nervous system neoplasms: malignant peripheral nerve sheath tumors as a paradigm for cross-species comparative oncogenomics. Am J Pathol 186:464–477 . doi: 10.1016/j.ajpath.2015.10.023LK - http://sfx.library.uu.nl/utrecht?sid=EMBASE&issn=15252191&id=doi:10.1016%2Fj.ajpath.2015.10.023&atitle=The+challenge+of+cancer+genomics+in+rare+nervous+system+neoplasms%3A+Malignant+peripheral+nerve+sheath+tumors+as+a+paradigm+for+cross-species+comparative+oncogenomics&stitle=Am.+J.+Pathol.&title=American+Journal+of+Pathology&volume=186&issue=3&spage=464&epage=477&aulast=Carroll&aufirst=Steven+L.&auinit=S.L.&aufull=Carroll+S.L.&coden=AJPAA&isbn=&pages=464-477&d 10.1016/j.ajpath.2015.10.023 PMC4816695 26740486

[35] Goldblum J, Weiss S, Folpe AL (2019) Enzinger and Weiss’s Soft Tissue Tumor, 7th Editio. Elsevier Ltd

[36] Le Guellec S, Decouvelaere A-V, Filleron T (2016). Malignant peripheral nerve sheath tumor is a challenging diagnosis: a systematic pathology review, immunohistochemistry, and molecular analysis in 160 patients from the French Sarcoma Group database. Am J Surg Pathol.

[37] Rodriguez FJ, Folpe AL, Giannini C, Perry A (2012). Pathology of peripheral nerve sheath tumors: diagnostic overview and update on selected diagnostic problems. Acta Neuropathol.

[38] Martinez AP, Fritchie KJ (2019). Update on peripheral nerve sheath tumors. Surg Pathol Clin.

[39] Röhrich M, Koelsche C, Schrimpf D, Capper D, Sahm F, Kratz A, Reuss J, Hovestadt V, Jones DTW, Bewerunge-Hudler M, Becker A, Weis J, Mawrin C, Mittelbronn M, Perry A, Mautner VF, Mechtersheimer G, Hartmann C, Okuducu AF, Arp M, Seiz-Rosenhagen M, Hänggi D, Heim S, Paulus W, Schittenhelm J, Ahmadi R, Herold-Mende C, Unterberg A, Pfister SM, von Deimling A, Reuss DE (2016). Methylation-based classification of benign and malignant peripheral nerve sheath tumors. Acta Neuropathol.

[40] Wasa J, Nishida Y, Tsukushi S, Shido Y, Sugiura H, Nakashima H, Ishiguro N (2010). MRI features in the differentiation of malignant peripheral nerve sheath tumors and neurofibromas. Am J Roentgenol.

[41] Ferner RE (2007). Neurofibromatosis 1. Eur J Hum Genet.

[42] Perez-Roman RJ, Shelby Burks S, Debs L, Cajigas I, Levi AD (2020). The risk of peripheral nerve tumor biopsy in suspected benign etiologies. Neurosurgery..

[43] Graham DS, Russell TA, Eckardt MA, Motamedi K, Seeger LL, Singh AS, Bernthal NM, Kalbasi A, Dry SM, Nelson SD, Elashoff D, Levine BD, Eilber FC (2019). Oncologic accuracy of image-guided percutaneous core-needle biopsy of peripheral nerve sheath tumors at a high-volume sarcoma center. Am J Clin Oncol.

[44] Spurlock G, Knight SJL, Thomas N, Kiehl TR, Guha A, Upadhyaya M (2010). Molecular evolution of a neurofibroma to malignant peripheral nerve sheath tumor (MPNST) in an NF1 patient: correlation between histopathological, clinical and molecular findings. J Cancer Res Clin Oncol.

[45] Derlin T, Tornquist K, Munster S (2013). Comparative effectiveness of 18F-FDG PET/CT versus whole-body MRI for detection of malignant peripheral nerve sheath tumors in neurofibromatosis type 1. Clin Nucl Med.

[46] Reinert CP, Schuhmann MU, Bender B, Gugel I, la Fougère C, Schäfer J, Gatidis S (2019). Comprehensive anatomical and functional imaging in patients with type I neurofibromatosis using simultaneous FDG-PET/MRI. Eur J Nucl Med Mol Imaging.

[47] Bhargava R, Parham DM, Lasater OE, Chari RS, Chen G, Fletcher BD (1997). MR imaging differentiation of benign and malignant peripheral nerve sheath tumors: use of the target sign. Pediatr Radiol.

[48] Ferner RE, Golding JF, Smith M, Calonje E, Jan W, Sanjayanathan V, O’Doherty M (2008). [18F]2-fluoro-2-deoxy-D-glucose positron emission tomography (FDG PET) as a diagnostic tool for neurofibromatosis 1 (NF1) associated malignant peripheral nerve sheath tumours (MPNSTs): a long-term clinical study. Ann Oncol.

[49] Warbey VS, Ferner RE, Dunn JT, Calonje E, O’Doherty MJ (2009). [18F] FDG PET/CT in the diagnosis of malignant peripheral nerve sheath tumours in neurofibromatosis type-1. Eur J Nucl Med Mol Imaging.

[50] Ahlawat S, Blakeley JO, Rodriguez FJ, Fayad LM (2019). Imaging biomarkers for malignant peripheral nerve sheath tumors in neurofibromatosis type 1. Neurology.

[51] Broski SM, Johnson GB, Howe BM, Nathan MA, Wenger DE, Spinner RJ, Amrami KK (2016). Evaluation of 18F-FDG PET and MRI in differentiating benign and malignant peripheral nerve sheath tumors. Skelet Radiol.

[52] Salamon J, Veldhoen S, Apostolova I, Bannas P, Yamamura J, Herrmann J, Friedrich RE, Adam G, Mautner VF, Derlin T (2014). F-18-FDG PET/CT for detection of malignant peripheral nerve sheath tumours in neurofibromatosis type 1: tumour-to-liver ratio is superior to an SUVmax cut-off. Eur Radiol.

[53] Schwabe M, Spiridonov S, Yanik EL, Jennings JW, Hillen T, Ponisio M, McDonald DJ, Dehdashti F, Cipriano CA (2019). How effective are noninvasive tests for diagnosing malignant peripheral nerve sheath tumors in patients with neurofibromatosis type 1? Diagnosing MPNST in NF1 patients. Sarcoma.

[54] Combemale P, Valeyrie-Allanore L, Giammarile F, Pinson S, Guillot B, Goulart DM, Wolkenstein P, Blay JY, Mognetti T (2014). Utility of 18F-FDG PET with a semi-quantitative index in the detection of sarcomatous transformation in patients with neurofibromatosis type 1. PLoS One.

[55] Cook GJR, Lovat E, Siddique M, Goh V, Ferner R, Warbey VS (2017). Characterisation of malignant peripheral nerve sheath tumours in neurofibromatosis-1 using heterogeneity analysis of (18)F-FDG PET. Eur J Nucl Med Mol Imaging.

[56] Well L, Salamon J, Kaul MG, Farschtschi S, Herrmann J, Geier KI, Hagel C, Bockhorn M, Bannas P, Adam G, Mautner VF, Derlin T (2019). Differentiation of peripheral nerve sheath tumors in patients with neurofibromatosis type 1 using diffusion-weighted magnetic resonance imaging. Neuro-Oncology.

[57] Sauter ER (2017). Reliable biomarkers to identify new and recurrent cancer. Eur J breast Heal.

[58] Frankel S, Smith GD, Donovan J, Neal D (2003) Screening for prostate cancer. Lancet (London, England) 361:1122–1128 10.1016/S0140-6736(03)12890-5 10.1016/S0140-6736(03)12890-5 12672328

[59] Chu GCW, Lazare K, Sullivan F (2018). Serum and blood based biomarkers for lung cancer screening: a systematic review. BMC Cancer.

[60] Hummel TR, Jessen WJ, Miller SJ, Kluwe L, Mautner VF, Wallace MR, Lazaro C, Page GP, Worley PF, Aronow BJ, Schorry EK, Ratner N (2010). Gene expression analysis identifies potential biomarkers of neurofibromatosis type 1 including adrenomedullin. Clin Cancer Res.

[61] Johansson G, Peng P-C, Huang P-Y, Chien HF, Hua KT, Kuo ML, Chen CT, Lee MJ (2014). Soluble AXL: a possible circulating biomarker for neurofibromatosis type 1 related tumor burden. PLoS One.

[62] Park S-J, Sawitzki B, Kluwe L, Mautner VF, Holtkamp N, Kurtz A (2013). Serum biomarkers for neurofibromatosis type 1 and early detection of malignant peripheral nerve-sheath tumors. BMC Med.

[63] Watson KL, Al Sannaa GA, Kivlin CM (2017). Patterns of recurrence and survival in sporadic, neurofibromatosis type 1-associated, and radiation-associated malignant peripheral nerve sheath tumors. J Neurosurg.

[64] Puffer RC, Graffeo CS, Mallory GW (2016). Brain metastasis from malignant peripheral nerve sheath tumors. World Neurosurg.

[65] Casali PG, Abecassis N, Aro HT (2018). Soft tissue and visceral sarcomas: ESMO-EURACAN Clinical Practice Guidelines for diagnosis, treatment and follow-up. Ann Oncol Off J Eur Soc Med Oncol.

[66] Ferner RE, Gutmann DH (2002). International consensus statement on malignant peripheral nerve sheath tumors in neurofibromatosis. Cancer Res.

[67] Nelson CN, Dombi E, Rosenblum JS, Miettinen MM, Lehky TJ, Whitcomb PO, Hayes C, Scott G, Benzo S, Widemann BC, Chittiboina P (2019) Safe marginal resection of atypical neurofibromas in neurofibromatosis type 1. J Neurosurg:1–11. 10.3171/2019.7.JNS191353 10.3171/2019.7.JNS191353 PMC8320705 31653805

[68] Wong WW, Hirose T, Scheithauer BW, Schild SE, Gunderson LL (1998). Malignant peripheral nerve sheath tumor: analysis of treatment outcome. Int J Radiat Oncol Biol Phys.

[69] Zehou O, Fabre E, Zelek L, Sbidian E, Ortonne N, Banu E, Wolkenstein P, Valeyrie-Allanore L (2013). Chemotherapy for the treatment of malignant peripheral nerve sheath tumors in neurofibromatosis 1: a 10-year institutional review. Orphanet J Rare Dis.

[70] Puffer RC, Marek T, Stone JJ, Raghunathan A, Howe BM, Spinner RJ (2018). Extensive perineural spread of an intrapelvic sciatic malignant peripheral nerve sheath tumor: a case report. Acta Neurochir.

[71] Martin E, Slooff W-BM, van Houdt WJ, van Dalen T, Verhoef C, Coert JH (2020). Oncological treatment considerations differ across surgical subspecialties treating malignant peripheral nerve sheath tumors: an international survey. Sarcoma.

[72] Clarkson PW, Griffin AM, Catton CN, O'Sullivan B, Ferguson PC, Wunder JS, Bell RS (2005). Epineural dissection is a safe technique that facilitates limb salvage surgery. Clin Orthop Relat Res.

[73] Gerrand CH, Wunder JS, Kandel RA, O’Sullivan B, Catton CN, Bell RS, Griffin AM, Davis AM (2001). Classification of positive margins after resection of soft-tissue sarcoma of the limb predicts the risk of local recurrence. J Bone Joint Surg (Br).

[74] Dunn GP, Spiliopoulos K, Plotkin SR, Hornicek FJ, Harmon DC, Delaney TF, Williams Z (2013). Role of resection of malignant peripheral nerve sheath tumors in patients with neurofibromatosis Type 1: clinical article. J Neurosurg.

[75] Mundinger GS, Prucz RB, Frassica FJ, Deune EG (2014). Concomitant upper extremity soft tissue sarcoma limb-sparing resection and functional reconstruction: assessment of outcomes and costs of surgery. Hand (N Y).

[76] Martin E, Dullaart MJ, van de Sande MAJ, et al (2019) Resuscitating extremities after soft tissue sarcoma resections: are functional reconstructions an overlooked option in limb salvage? A systematic review. Eur J Surg Oncol in press: 10.1016/j.ejso.2019.05.024 10.1016/j.ejso.2019.05.024 31178300

[77] Martin E, Dullaart MJ, Verhoef C, Coert JH (2020). A systematic review of functional outcomes after nerve reconstruction in extremity soft tissue sarcomas: a need for general implementation in the armamentarium. J Plast Reconstr Aesthet Surg.

[78] Spiliopoulos K, Williams Z (2011). Brachial plexus reconstruction following resection of a malignant peripheral nerve sheath tumor: case report. Neurosurgery.

[79] Costales JR, Socolovsky M, Sanchez Lazaro JA, Alvarez Garcia R (2019). Peripheral nerve injuries in the pediatric population: a review of the literature. Part I: traumatic nerve injuries. Childs Nerv Syst.

[80] Slump J, Hofer SOP, Ferguson PC, Wunder JS, Griffin AM, Hoekstra HJ, Bastiaannet E, O'Neill AC (2018). Flap reconstruction does not increase complication rates following surgical resection of extremity soft tissue sarcoma. Eur J Surg Oncol.

[81] Barwick WJ, Goldberg JA, Scully SP, Harrelson JM (1992). Vascularized tissue transfer for closure of irradiated wounds after soft tissue sarcoma resection. Ann Surg.

[82] J. K, A. G, M. T, et al (2014) Radiation therapy in management of sporadic and neurofibromatosis type 1-associated malignant peripheral nerve sheath tumors. Front Oncol 4: . doi: 10.3389/fonc.2014.00324LK - http://sfx.library.uu.nl/utrecht?sid=EMBASE&issn=2234943X&id=doi:10.3389%2Ffonc.2014.00324&atitle=Radiation+therapy+in+management+of+sporadic+and+neurofibromatosis+type+1-associated+malignant+peripheral+nerve+sheath+tumors&stitle=Front.+Oncol.&title=Frontiers+in+Oncology&volume=4&issue=NOV&spage=&epage=&aulast=Kahn&aufirst=Jenna&auinit=J.&aufull=Kahn+J.&coden=&isbn=&pages=-&date=2014&auinit1=J&auinitm= 10.3389/fonc.2014.00324 PMC4233912 25452937

[83] Ferrari A, Bisogno G, Carli M (2007). Management of childhood malignant peripheral nerve sheath tumor. Paediatr Drugs.

[84] Kaushal A, Citrin D (2008). The role of radiation therapy in the management of sarcomas. Surg Clin North Am.

[85] Davis A, O’Sullivan B, Turcotte R (2005). Late radiation morbidity following randomization to preoperative versus postoperative radiotherapy in extremity soft tissue sarcoma. Radiother Oncol.

[86] O’Sullivan B, Davis AM, Turcotte R (2002). Preoperative versus postoperative radiotherapy in soft-tissue sarcoma of the limbs: a randomised trial. Lancet (London, England).

[87] Clark MA, Fisher C, Judson I, Thomas JM (2005). Soft-tissue sarcomas in adults. N Engl J Med.

[88] Hawkins DS, Spunt SL, Skapek SX (2013). Children’s Oncology Group’s 2013 blueprint for research: soft tissue sarcomas. Pediatr Blood Cancer.

[89] Pratt CB, Pappo AS, Gieser P, Jenkins JJ, Salzberg A, Neff J, Rao B, Green D, Thomas P, Marcus R, Parham D, Maurer H (1999). Role of adjuvant chemotherapy in the treatment of surgically resected pediatric nonrhabdomyosarcomatous soft tissue sarcomas: a Pediatric Oncology Group Study. J Clin Oncol Off J Am Soc Clin Oncol.

[90] Grobmyer SR, Reith JD, Shahlaee A, Bush CH, Hochwald SN (2008). Malignant peripheral nerve sheath tumor: molecular pathogenesis and current management considerations. J Surg Oncol.

[91] von Mehren M, Randall RL, Benjamin RS, Boles S, Bui MM, Conrad EU, Ganjoo KN, George S, Gonzalez RJ, Heslin MJ, Kane JM, Koon H, Mayerson J, McCarter M, McGarry SV, Meyer C, O'Donnell RJ, Pappo AS, Paz IB, Petersen IA, Pfeifer JD, Riedel RF, Schuetze S, Schupak KD, Schwartz HS, Tap WD, Wayne JD, Bergman MA, Scavone J (2016). Soft tissue sarcoma, Version 2.2016, NCCN Clinical Practice Guidelines in Oncology. J Natl Compr Cancer Netw.

[92] Higham CS, Steinberg SM, Dombi E, Perry A, Helman LJ, Schuetze SM, Ludwig JA, Staddon A, Milhem MM, Rushing D, Jones RL, Livingston M, Goldman S, Moertel C, Wagner L, Janhofer D, Annunziata CM, Reinke D, Long L, Viskochil D, Baker L, Widemann BC (2017). SARC006: phase II trial of chemotherapy in sporadic and neurofibromatosis type 1 associated chemotherapy-naive malignant peripheral nerve sheath tumors. Sarcoma.

[93] Dombi E, Baldwin A, Marcus LJ, Fisher MJ, Weiss B, Kim AR, Whitcomb P, Martin S, Aschbacher-Smith LE, Rizvi TA, Wu J, Ershler R, Wolters P, Therrien J, Glod J, Belasco JB, Schorry E, Brofferio A, Starosta AJ, Gillespie A, Doyle AL, Ratner N, Widemann BC (2016). Activity of selumetinib in neurofibromatosis type 1-related plexiform neurofibromas. N Engl J Med.

[94] Tap WD, Jones RL, Van Tine BA (2016). Olaratumab and doxorubicin versus doxorubicin alone for treatment of soft-tissue sarcoma: an open-label phase 1b and randomised phase 2 trial. Lancet (London, England).

[95] Robertson KA, Nalepa G, Yang F-C, Bowers DC, Ho CY, Hutchins GD, Croop JM, Vik TA, Denne SC, Parada LF, Hingtgen CM, Walsh LE, Yu M, Pradhan KR, Edwards-Brown MK, Cohen MD, Fletcher JW, Travers JB, Staser KW, Lee MW, Sherman MR, Davis CJ, Miller LC, Ingram DA, Clapp DW (2012). Imatinib mesylate for plexiform neurofibromas in patients with neurofibromatosis type 1: a phase 2 trial. Lancet Oncol.

[96] Chugh R, Wathen JK, Maki RG, Benjamin RS, Patel SR, Myers PA, Priebat DA, Reinke DK, Thomas DG, Keohan ML, Samuels BL, Baker LH (2009). Phase II multicenter trial of imatinib in 10 histologic subtypes of sarcoma using a bayesian hierarchical statistical model. J Clin Oncol.

[97] Ferguson MJ, Rhodes SD, Jiang L, Li X, Yuan J, Yang X, Zhang S, Vakili ST, Territo P, Hutchins G, Yang FC, Ingram DA, Clapp DW, Chen S (2016). Preclinical evidence for the use of sunitinib malate in the treatment of plexiform neurofibromas. Pediatr Blood Cancer.

[98] Kim A, Dombi E, Tepas K, Fox E, Martin S, Wolters P, Balis FM, Jayaprakash N, Turkbey B, Muradyan N, Choyke PL, Reddy A, Korf B, Widemann BC (2013). Phase I trial and pharmacokinetic study of sorafenib in children with neurofibromatosis type I and plexiform neurofibromas. Pediatr Blood Cancer.

[99] Ahsan S, Ge Y, Tainsky MA (2016). Combinatorial therapeutic targeting of BMP2 and MEK-ERK pathways in NF1-associated malignant peripheral nerve sheath tumors. Oncotarget.

[100] Albritton KH, Rankin C, Coffin CM, Ratner N, Budd GT, Schuetze SM, Randall RL, Declue JE, Borden EC (2006). Phase II study of erlotinib in metastatic or unresectable malignant peripheral nerve sheath tumors (MPNST). J Clin Oncol.

[101] Maki RG, D’Adamo DR, Keohan ML (2009). Phase II study of sorafenib in patients with metastatic or recurrent sarcomas. J Clin Oncol.

[102] Schuetze SM, Wathen JK, Lucas DR, Choy E, Samuels BL, Staddon AP, Ganjoo KN, von Mehren M, Chow WA, Loeb DM, Tawbi HA, Rushing DA, Patel SR, Thomas DG, Chugh R, Reinke DK, Baker LH (2016). SARC009: phase 2 study of dasatinib in patients with previously treated, high-grade, advanced sarcoma. Cancer.

[103] Martin E, Lamba N, Flucke UE, Verhoef C, Coert JH, Versleijen-Jonkers YMH, Desar IME (2019). Non-cytotoxic systemic treatment in malignant peripheral nerve sheath tumors (MPNST): a systematic review from bench to bedside. Crit Rev Oncol Hematol.

[104] Widemann BC, Meyer CF, Cote GM, Chugh R, Milhem MM, van Tine BA, Kim AR, Turpin B, Dombi E, Jayaprakash N, Okuno SH, Helman LJ, Onwudiwe N, Steinberg SM, Reinke DK, Cichowski K, Perentesis JP (2016). SARC016: phase II study of everolimus in combination with bevacizumab in sporadic and neurofibromatosis type 1 (NF1) related refractory malignant peripheral nerve sheath tumors (MPNST). J Clin Oncol.

[105] Tsuchida T, Otsuka H, Niimura M, Inoue Y, Kukita A, Hashimoto Y, Seyama Y, Yamakawa T (1984). Biochemical study on gangliosides in neurofibromas and neurofibrosarcomas of Recklinghausen’s disease. J Dermatol.

[106] Yu AL, Gilman AL, Ozkaynak MF, London WB, Kreissman SG, Chen HX, Smith M, Anderson B, Villablanca JG, Matthay KK, Shimada H, Grupp SA, Seeger R, Reynolds CP, Buxton A, Reisfeld RA, Gillies SD, Cohn SL, Maris JM, Sondel PM, Children's Oncology Group (2010). Anti-GD2 antibody with GM-CSF, interleukin-2, and isotretinoin for neuroblastoma. N Engl J Med.

[107] D’Angelo SP, Mahoney MR, Van Tine BA (2018). Nivolumab with or without ipilimumab treatment for metastatic sarcoma (Alliance A091401): two open-label, non-comparative, randomised, phase 2 trials. Lancet Oncol.

